# Irisin attenuates angiotensin II-induced atrial fibrillation and atrial fibrosis via LOXL2 and TGFβ1/Smad2/3 signaling pathways

**DOI:** 10.22038/IJBMS.2023.68639.14967

**Published:** 2023

**Authors:** Yingbiao Wu, Jun Luo, Xiang Song, Wei Gu, Saihua Wang, Shuwen Hao, Zhiwu Dong, Zhongping Ning

**Affiliations:** 1 Department of Cardiology, Shanghai University of Medicine & Health Sciences affiliated Zhoupu Hospital, Shanghai 201318, China; 2 Department of Cardiology, People’s Hospital of Shache County, Xinjiang, 844700, China; # These authors contributed equally to this work

**Keywords:** Angiotensin II, Atrial fibrillation, Atrial fibrosis, Irisin, Lysyl oxidase (LOX)-like-2, Transforming growth factor-beta/Smad

## Abstract

**Objective(s):**

Irisin was reported as a cardioprotective and anti-oxidative effector, while the effect on atrial fibrosis is unknown. The current research examined irisin’s function in atrial fibrillation (AF); atrial fibrosis brought on by Ang II can be suppressed, thus lessening the risk of developing AF.

**Materials and Methods:**

246 individuals were enrolled in the present case-control study. Chinese AF patients (n=126), 83 of whom were paroxysmal AF (PAF), 43 patients with persistent AF (PeAF), and 120 healthy controls. Saline or Ang II (2.0 mg/kg/day) was subcutaneously injected into healthy male C57BL/6 mice for four weeks. Once daily for four weeks, intraperitoneal injections of exogenous irisin (500 g/kg/day) were administered.

**Results:**

In comparison to PAF patients and healthy controls (all P<0.05), PeAF patients had significantly higher rates of heart failure (HF), large left atrial size (LAD), hypertrophic protein B-type natriuretic peptide (BNP), malondialdehyde (MDA), tumor necrosis factor-α (TNF-α), interleukin-6 (IL-6), C-terminal telopeptide of type I collagen (CTX-I), and transforming growth factor beta-1 (TGF-β1), while superoxide dismutase (SOD) level was low. Expression of irisin was decreased in AF patients’ serum and Ang II-infused mice. Exogenous irisin dramatically reduced apoptosis, atrial fibrosis, atrial inflammation, and the susceptibility to AF caused by Ang II. In the atrial tissue, irisin inhibited Ang II-induced fibroblast transdifferentiation, LOXL2, TGF-β1, collagen production, and phosphorylation of Smad2/3.

**Conclusion:**

The study results speculated that irisin could be a potential AF target, and it inhibited atrial fibrosis and significantly impaired increased AF susceptibility through inactivation of LOXL2 and the TGF-β/Smad pathway.

## Introduction

Atrial fibrillation (AF), which is the most common heart arrhythmia, elevates the heart attack risk and heart failure (HF) and, as a result, increases cardiac disease-related morbidity and mortality (1, 2). The occurrence of AF is dramatically increased with age, and most of the patients are aged ≥65 years (3). The increasing incidence of AF may likely expand the financial problems of patients and families. Consequently, AF could be a potential threat to global human health.

In the past, several studies explored whether AF has complex mechanisms associated with different ailments, such as hypertension, coronary artery disease, diabetes, and aging (3). Triggers and substrates associated with AF pathogenesis are more likely interconnected. Atrial fibrosis is the most prominent type of substrate for atrial remodeling, where atrial remodeling plays a critical role in AFs pathogenesis (4, 5). The extracellular matrix (ECM) protein accumulation, especially collagen, is the most prominent expression of atrial fibrosis. ECM protein deposition elevates tissue stiffness and induces diastolic cardiac dysfunction.

A versatile cytokine is called transforming growth factor beta-1 (TGF- β1) with potential inflammatory and regulatory activity (6). Type I and type II receptors of TGF-β1 attach to them to activate phosphorylation reactions that elevate Smad proteins 2, 3, and 4 inactivation. It takes place just before the Smad complex is translocated into the nucleus and inhibits the synthesis of the profibrotic protein connective tissue growth factor and collagen (1, 7). Previous research reported the TGF-β1 and collagen expression up-regulated by Angiotensin (Ang II) *)* and *in vivo*. In addition, modulation of TGF-β1 by Ang II may influence atrial fibrosis and AF (8-11). Thus, it could be a possible therapeutic approach to prevent atrial fibrosis induced by Ang II inhibition of the TGF-β1/Smads pathway.

A copper-dependent amine oxidase is part of the LOX family, including lysyl oxidase (LOX)-like-2 (LOXL2), and could be associated with matrix remodeling and fibrogenesis (12). LOXL2 may be connected to extracellular matrix deposition, epithelial-mesenchymal transition, and the development and incidence of fibrosis-related disorders (13). Recently it was reported that LOXL2 is associated with cardiac fibrosis in HF and up-regulated in human heart diseases in the cardiac interstitium. In addition, LOXL2 levels might be involved in crosslinking collagen deposition and cardiac disorders. However, LOXL2 levels were increased in the serum of people with HF, and it was speculated that LOXL2 could have pathogenic activity on cardiac fibrosis and HF (14, 15).

 Irisin is a novel myokine, first reported in 2012. It is proteolytically cleavaged of fibronectin type III domain containing 5 (FNDC5) (16, 17). Irisin is a hormone with a metabolic function that controls non-alcoholic fatty liver disease, type 2 diabetes, and obesity. Moreover, it could reduce inflammation and be crucial for protecting mitochondria (18-20). Recently it was reported that irisin promoted neurologically generated neurotrophic factors secretion and showed a neuroprotective effect in Alzheimer’s disease (21). In addition, a lower level of irisin may be involved in endothelial dysfunction and atherosclerosis (22). However, the function of irisin in atrial fibrosis is largely unknown. 

 The current study performed a clinical study on humans and then examined Irisin’s function in Ang II-induced mice with AF vulnerability and atrial fibrosis and its potential effects on the LOXL2 and TGFβ1/Smad2/3 signaling pathways. 

## Materials and Methods

***Study population***

This observational study included 126 patients with atrial fibrillation admitted to the Department of Cardiology at Zhoupu Hospital from January 2018 to December 2020. The diagnosis of non-valvular atrial fibrillation was based on the auscultation of superior clinical doctors and the report of 12-limb lead ECG, 24-hour ambulatory ECG, and echocardiography. The AF patients were categorized into two groups: the paroxysmal AF group (PAF; n = 83) and the persistent AF group (PeAF; n = 43). One hundred twenty (120) healthy controls were included for the same period, and there were no statistically significant differences in sex or age between the healthy controls and AF groups (*P*>0.05). The age range of patients was 46 to 85 years old. The exclusion criteria were as follows: (1) patients with valvular heart disease, vein thrombosis, cardiomyopathy, and severe heart failure; (2) patients with hyperthyroidism and hypothyroidism, (3) patients with myocardial infarction within half a year and cardiac surgery within one month; (4) patients with ischemic stroke and transient ischemic attack (TIA); (5) patients with cancer, severe liver, kidney, and coagulation abnormalities. 

***Clinical data and laboratory tests***

Peripheral blood of 5 ml was collected from fasting patients and healthy controls in the morning with EDTA anticoagulant tube, the blood sample was kept at room temperature for 2 hr or 4 °C overnight, and then centrifuged at 1500 × g for 15 min, the supernatant was taken and stored at -80 °C for testing. The basic data of all subjects were recorded, including name, gender, age, personal history, previous history, family history, and laboratory tests, including total cholesterol (TC), triglyceride (TG), low-density lipoprotein (LDL-C), and high-density lipoprotein (HDL-C), which were measured by the automatic biochemical analyzer in the Central Laboratory of our hospital. Left atrial size (LAD) was collected from echocardiography.

***Animals and treatment***

Healthy male C57BL/6 mice (8 weeks old, 20–25 g) were purchased from Shanghai SLRC Laboratory Animal Co, Ltd. (Shanghai, China) and kept in an SPF condition with 22–24 °C temperature and 60–65% relative humidity. Mice were subcutaneously infused with saline or Ang II (2.0 mg/kg/day) using osmotic mini-pumps for 4 weeks. The exogenous irisin protein (500 μg/kg/day) (HY-P72534, MedChemExpress, USA) or saline was administered intraperitoneally for 4 weeks. The Animal Care and Use Committee of Shanghai Pudong Zhoupu Hospital approved the study.

***Arrhythmia inducibility and duration by transesophageal burst pacing***

On day 28 after Ang II infusion, mice were anesthetized by 1% pentobarbital sodium intraperitoneal injection, and the body temperature was kept at 37 °C during the procedure. An 8-electrode catheter (Japan Lifeline, Tokyo, Japan) was inserted near the left atrium in the esophagus to record an electrocardiogram (ECG). AF was defined as a rapid, irregular atrial rhythm with irregular R-R intervals of 1 sec. A physiopharmacology electronic stimulator was applied to stimulate three trains of 2 sec bursts to test the inducibility of AF. The parameters were set as follows: voltage: 20 V; current: 4 mA; wave width: 6 ms; interval: 20 ms. The incidence and duration of AF were measured from the end of burst pacing to the first P-wave.

***Sample collection***

After ECG recording, mice were anesthetized with 1.25% tribromoethanol (0.02 ml/g) for 30 min. After deep anesthesia, the mice were killed by cervical dislocation. Some tissue was snap-frozen in liquid nitrogen for molecular biology analysis and another tissue was fixed in 4% paraformaldehyde for histological studies.

***Tissue histology and immunohistochemistry***

The atrial tissue was fixed with 4% paraformaldehyde, paraffin-embedding, and cutting embedded paraffin into 5 μm serial sections. Slides were stained with Masson’s trichrome or incubated with rabbit polyclonal antibodies against α-SMA antibody (1:400; ab5694; Abcam, UK) or LOXL2 (1:200; ab96233; Abcam, UK) at 37 °C for 20 min, followed by biotinylated HRP conjugated secondary antibody. Immunofluorescence staining was performed using TUNEL and DAPI double staining. The slides were washed and counter-stained. Images were photographed from ten random fields per sample (x200 magnification; Nikon, Tokyo, Japan) and were analyzed using ImageJ (NIH, Bethesda, MD, USA). The fibrosis extent was evaluated by the ratio of fibrotic area to normal myocardium and was reported as collagen volume fraction.

***Serum cytokines measurement assay***

Enzyme-Linked Immunosorbent Assays (ELISA) were used to determine the cytokine levels in the mice’s serum. The blood sample was obtained from the caudal vein of mice after four weeks of Ang II infusion, and serum was isolated and stored at -80 °C. Levels of human irisin (DY9420-05 ), mice irisin (FT-P9S1398X), TNF-α (MTA00B), IL-6 (M6000B), and TGF-β (DB100B) were measured with commercial ELISA kits (R&D Systems, Minneapolis, MN, USA), CTX-I was measured by ml028171, Shanghai Enzyme-linked Biotechnology Co., Ltd, China.

***RT-qPCR***

Total RNA was extracted from ventricular tissue using TRIzol reagent (Invitrogen, USA) and quantified by a NanoDrop^TM^ (Thermo Fisher Scientific). The quantity and purity of total RNA were measured by a NanoDrop^TM^ (Thermo Fisher Scientific). The RNA was reversely transcribed into complementary DNA (cDNA), and the genomic DNA was digested by DNase I (Takara Bio, China). The real-time quantitative polymerase chain reaction was carried out by a Verso SYBR Green 1-Step QRT-PCR kit (Thermo Fisher Scientific). Primer sequences were as follows: FNDC5 (forward: 5-AAG TGG TCA TTG GCT TTG C-3′; reverse: 5′-GTT GTT ATT GGG CTC GTG T-3′); COL1A1 (forward: 5′-TAA AGG GTC ATC GTG GCT TC-3′; reverse: 5′-GAC GGC TGA GTA GGG AAC AC-3′); COL3A1 (forward: 5′-GCG GAA TTC CTG GAC CAA AAG GTG ATG CTG-3′; reverse: 5′-GCG GGA TCC GAG GAC CAC GTT CCC CAT TAT-3′); GAPDH (forward: 5′-ACT CCA CTC ACG GCA AAT TC-3′; reverse: 5′-TCT CCA TGG TGG TGA AGA CA-3′). Experiments were carried out in triplicate plate wells with at least three replicates per PCR reaction. The expression of GAPDH mRNA was used as an internal control.

***Western blotting***

Proteins were isolated from ventricular tissue using lysis buffer and quantified by a BCA assay kit (Beyotime). Total protein (50 μg) was separated by SDS-PAGE and transferred onto PVDF membranes. With 5% skimmed milk, the membranes were blocked and incubated with primary and secondary antibodies against LOXL2 (1:500, sc-293427, mouse monoclonal, Santa Cruz); FNDC5 (1:1000, ab131390), Collagen I (1:500, ab255809), Collagen III (1:500, ab7778), TGF-β1( 1:1000; ab179695), p-Smad2 (1:500, ab188334), p-Smad3 (1:500, ab52903), Smad2/3 (1:1000, ab202445), and GAPDH (1:1000, ab9485) (Abcam, Cambridge, UK). After washing with the Tris-buffered saline and Tween-20 mixture, membranes were incubated with horseradish peroxidase-conjugated secondary antibody for two hours at room temperature. Protein bands were identified with ECL reagents (Amersham Biosciences, UK). GAPDH was used as an internal control, and the protein bands were analyzed by ImageJ software.

***Statistical analysis***

Our study expressed data as mean ± SD and analyzed it by SPSS 19.0 statistical software. A Student t-test was applied to compare the difference between the two groups, and one-way ANOVA followed by Tukey’s *post hoc* test was carried out to compare every two groups among different groups. The Pearson correlation test was applied to analyze the correlation between continuous variables among AF patients. *P*<0.05 was regarded as a significant criterion for the difference.

***Baseline characteristics***

A total of 126 AF patients were included in this study, with 69 males and 57 females (mean age 66.37±9.25 years). Clinical, echocardiographic, and biochemical data from all AF patients were examined, and comparable information is presented in Table 1. Between AF patients and healthy controls, there was no discernible difference in age or gender. However, AF patients had higher body mass index (BMI), larger left atrial size (LAD), higher serum MDA, TNF-α, IL-6, CTX-I, TGF-β1, and lower serum SOD compared with healthy controls (all *P*<0.05). There were 126 individuals with atrial fibrillation (AF), 83 of them had paroxysmal atrial fibrillation (PAF), and 43 had persistent atrial fibrillation (PeAF). PAF patients contrasted with PeAF patients had a significantly higher incidence of heart failure (HF), larger LAD, higher serum levels of BNP, MDA, TNF-α, IL-6, CTX-I, and TGF-β1, and lower levels of SOD (all *P*<0.05) (Table 2).

***Serum irisin concentrations were reduced in AF patients***

**Figure 1:**
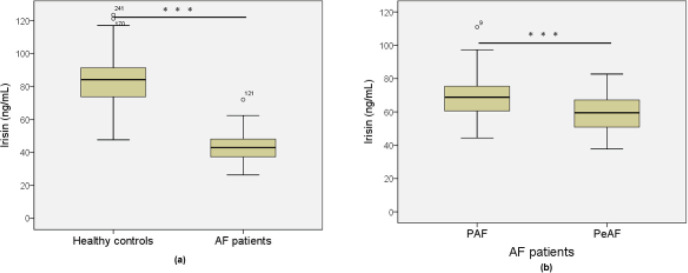
Serum irisin levels in AF patients and controls. (a) Serum irisin levels are higher in AF patients (n = 207) than in healthy controls (n = 160) (P<0.001). (b) Serum irisin levels are higher in the PeAF (n = 76) than in the PAF (n = 131). ***P<0.001

PAF: Paroxysmal atrial fibrillation; PeAF: Persistent atrial fibrillation

We measured AF patients’ serum irisin concentrations by ELISA assay. Serum irisin levels in AF patients were substantially lower (65.42±12.90 ng/ml) than in healthy controls (83.58±14.65 ng/ml) (*P*<0.001) (Figure 1A). In AF patients, serum irisin level was potentially reduced in PeAF (58.24±11.42 ng/ml) versus PAF (69.14±12.08 ng/ml) (*P*<0.001) (Figure 1B). 

***Correlations of serum irisin with clinical characteristics of AF patients***

We used a Pearson correlation test to ascertain the relationships between serum irisin and clinical and biochemical characteristics in AF patients. Serum irisin showed a significant positive correlation with SOD and a negative correlation with LAD, BNP, MDA, TNF-α, IL-6, CTX-I, and TGF-β1 (Table 3).

***Irisin was up-regulated in Ang II-induced mice***

**Figure 2:**
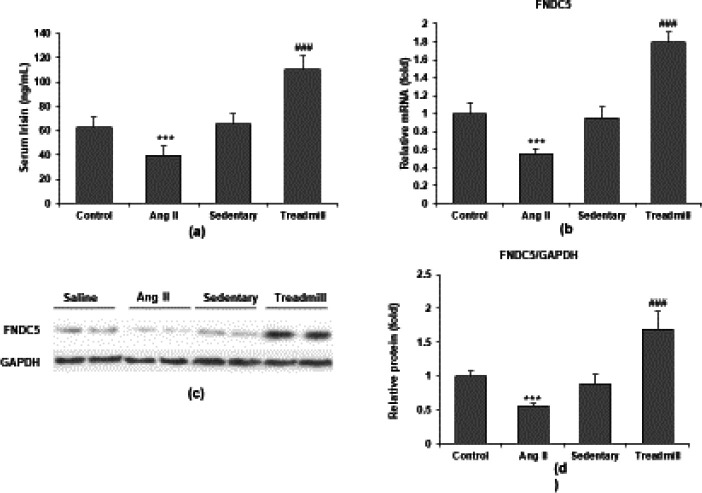
Irisin expression is decreased in Ang II-induced mice and increased by aerobic exercise. Mice were infused with saline or Ang II (2000 ng/kg/min) using osmotic mini-pumps for four weeks or kept in a sedentary state and subjected to aerobic exercise for four weeks. (a) Irisin protein levels in serum are significantly reduced in Ang-II-induced compared with control mice and are significantly increased in mice with aerobic exercise compared with sedentary mice. (b) mRNA levels of irisin precursor FNDC5 are determined in the atria muscle by RT-qPCR. (c) Representative bands of FNDC5 protein in atria muscle of mice by western blot. (d) Quantification of FNDC5 protein levels. Data are presented as mean ± SD (n=8 in each group). ***P<0.001 vs saline group; ###*P*<0.001 vs sedentary group

An ELISA assay was carried out in the mice serum that had Ang-II injected constantly or subjected to aerobic exercise (treadmill) for four weeks to investigate the expression of irisin and its precursor in atrial tissue. Serum irisin concentration was significantly reduced after Ang-II infusion and increased after aerobic exercise (Figure 2A). To explore the potential origin of serum irisin, we measured the mRNA and protein levels of irisin in the mice’s atrial tissue using RT-qPCR and western blot. In the left atrium of mice, Ang II infusion substantially decreased irisin mRNA and protein expression (both *P*<0.05). However, the expression of irisin mRNA and protein in atrial tissue was significantly increased by aerobic exercise (both *P*<0.05) (Figure 2B–2D).

***Irisin attenuated Ang II-induced AF in mice***

**Figure 3:**
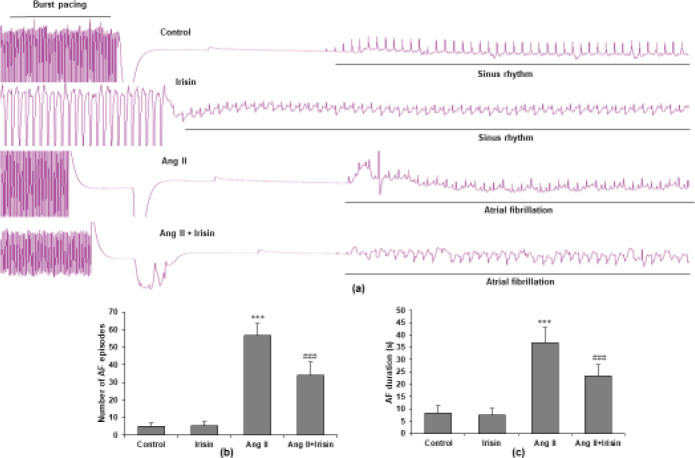
Irisin attenuates Ang II-induced susceptibility in AF. (a) For four weeks, representative atrial electrogram recordings of mice infused with saline, irisin, Ang II, and Ang II + irisin. (b) Numbers of AF episodes in mice. (c) Total AF duration. Data are presented as mean ± SD (n=8 in each group). ***P<0.001 vs saline group; ###*P*<0.001 vs Ang II group

Mice were injected with Ang II with or without irisin (500 g/kg/day) for 28 days to study the impact of exogenous irisin on the heart’s rhythm and susceptibility to AF. Then, we recorded the electrocardiogram followed by transesophageal fast atrial pacing. A typical atrial fibrillation attack was shown in AF mice, which can be attenuated by irisin treatment (Figure 3A). Ang II enhanced the inducibility of AF, according to quantification analysis, and irisin therapy significantly decreased Ang II-triggered AF inducibility (*P*<0.05) (Figure 3B). Moreover, irisin also reduced increased total AF length brought on by Ang II (*P*<0.05) (Figure 3C).

***Irisin attenuated Ang II-induced apoptosis and inflammation in atrial tissue***

**Figure 4:**
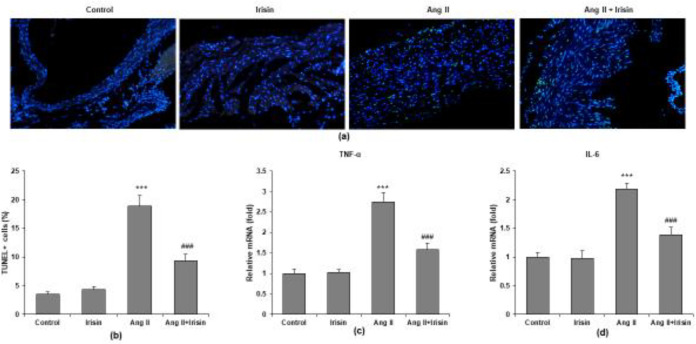
Irisin inhibits Ang II-induced atrial apoptosis and inflammation. (a) Representative image of TUNEL staining in atrial tissues of mice. (b) Quantification of TUNEL-positive cells in atrial tissues. RT-qPCR was carried out to determine the mRNA expression of (c) TNF-α and (d) IL-6 in atrial tissues. n = 8 mice per group. ***P<0.001 vs control group; ###*P*<0.001 vs Ang II group

We used TUNEL staining to determine how irisin affected atrial tissue apoptosis and inflammation. We demonstrated the reduction of atrial apoptosis caused by Ang II in mice treated with irisin (green fluorescence in the atrial tissue of mice) (Figure 4A). When irisin mice were compared with Ang II mice, quantitative analysis revealed that the TUNEL-positive cells were considerably lower in the irisin mice (Figure 4B). Furthermore, RT-qPCR showed two pro-inflammatory cytokines, TNF-α and IL-6, had considerably lower mRNA levels in the irisin mice than in the Ang II mice (Figure 4C, 4D).

***Irisin attenuated Ang II-induced atrial fibrosis***

**Figure 5:**
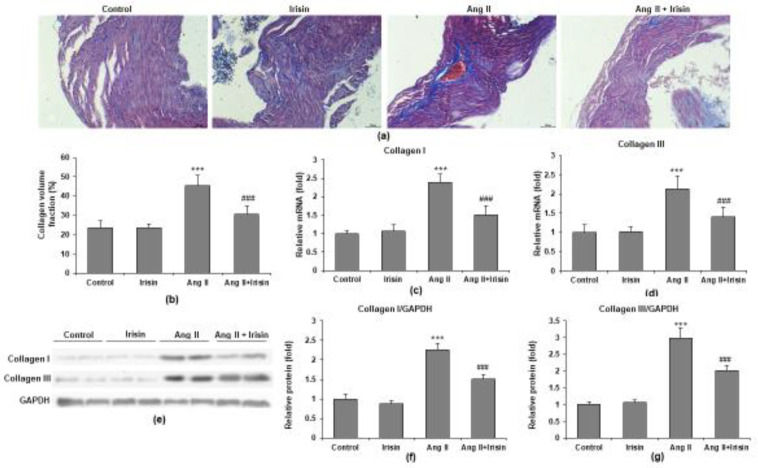
Irisin suppresses Ang II-induced atrial fibrosis. (a) Representative images of atrial fibrosis, which was stained with Masson trichrome. (b) Quantification of collagen volume fraction (%) is used to evaluate the fibrotic area. RT-qPCR analyses show (c) Collagen I and (d) Collagen III mRNA levels in atrial tissues. (e) Representative bands of proteins in atria muscle of mice by western blot. (f) Collagen I and (g) Collagen III proteins were quantified. n = 8 mice per group. ***P<0.001 vs control group; ##*P*<0.01, ###*P*<0.001 vs Ang II group

Irisin’s impact was noticeably decreased, whereas Ang II therapy greatly enhanced the atrial fibrotic region (Figure 5A, 5B). Additionally, RT-qPCR demonstrated that Ang II elevated the mRNA expression of two fibrotic markers, collagen I and III, in the mice atrial tissue, which was noticeably decreased by irisin administration (both *P*<0.05) (Figure 5C, 5D). The western blot analysis showed that collagen I and III protein expression in atrial tissue was also significantly reduced by irisin in mice with Ang II infusion (both *P*<0.05) (Figure 5E–5G).

***Irisin antagonist suppressed fibroblast transdifferentiation in atrial tissue***

**Figure 6:**
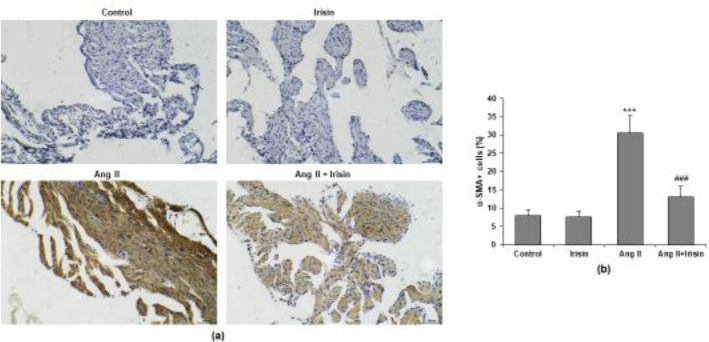
Irisin inhibited the fibroblast transdifferentiation of atrial tissue in Ang II-treated mice. (a) Immunohistochemistry image of α-SMA. (b) Quantification of the percentage of α-SMA-positive cells. n = 8 mice per group. ***P<0.001 vs control group; ###*P*<0.001 vs Ang II group

To investigate the role of irisin on fibroblast transdifferentiation, we performed immunohistochemistry to detect a myofibroblast marker expression. Ang II infusion enhanced the amount of α-SMA staining in the atrial tissue, but it was attenuated by irisin treatment (Figure 6A). Irisin significantly decreased the quantity of α-SMA-positive myofibroblasts in the atrial tissue of mice infused with Ang II, according to quantitative analysis (*P*<0.05) (Figure 6B).

***Irisin suppressed the expression of LOXL2 and TGFβ/Smad pathway***

**Figure 7:**
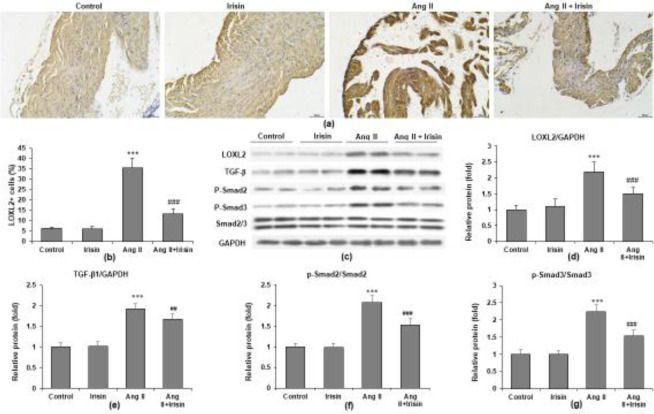
Irisin suppressed the expression of LOXL2 and TGFβ/Smad pathway in Ang II-treated mice. (a) Representative images of LOXL2 by immunohistochemistry. (b) Quantification of the percentage of LOXL2-positive cells. (c) Representative protein bands by western blot. Proteins are quantified for (d) LOXL2 (normalized to GAPDH), (e) TGFβ1 (normalized to GAPDH), (f) p-Smad2 (normalized to Smad2/3), and (g) p-Smad3 (normalized to Smad2/3). n = 8 mice per group. ***P<0.001 vs saline group; ##*P*<0.01, ###P<0.001 vs Ang II group

Immunohistochemistry was carried out to evaluate the expression of LOXL2. Irisin treatment enhanced the staining intensity of LOXL2, and Ang II infusion enhanced the quantity of LOXL2-positive cells in mice atrial tissue (Figure 7A, 7B). Western blotting analysis was used to assess the protein levels of LOXL2 and the TGFβ/Smad pathway (Figure 7C). The Ang II-induced rise in LOXL2 protein expression was dramatically reduced by irisin (Figure 7D), TGFβ1 (Figure 7E), p-Smad2 (Figure 7F), and p-Smad3 (Figure 7G) in the atrial tissue of mice.

**Table 1:** General clinical data of healthy controls and atrial fibrillation patients

Variables	Healthy controls (n=120)	AF patients (n=126)	T or χ2	*P*-value
Age (year)	65.94±11.90	66.37±9.25	0.316	0.752
Gender (male)	69 (57.5%)	69 (54.8%)	0.187	0.665
BMI (kg/m2)	24.23±1.71	25.28±2.31	4.068	<0.001
LAD (mm)	30.94±2.84	42.97±3.33	30.503	<0.001
MDA (mmol/ml)	4.07±0.75	7.30±0.92	30.261	<0.001
SOD (U/ml)	192.08±14.49	139.34±13.91	29.135	<0.001
TNF-α (ng/ml)	25.16±3.24	59.30±6.74	51.033	<0.001
IL-6 (pg/ml)	116.70±17.17	198.79±27.63	28.135	<0.001
CTX-I (ng/ml)	0.56±0.09	1.39±0.17	49.093	<0.001
TGF-β1 (ng/ml)	9.29±1.85	19.22±2.93	31.919	<0.001

Significance, *P*<0.05; comparing AF patients with healthy controls. Data are expressed as mean ± standard deviation (SD) for quantitative variables and expressed as cases and percentages for category variables. T-test or chi-square (χ2) test was used to test for significant differences

AF: Atrial fibrillation; BMI: Body mass index; LAD: Left atrial diameter; TNF-α: Tumor necrosis factor-α; IL-6: Interleukin-6; MDA: Malondialdehyde; SOD: Superoxide dismutase

**Table 2:** Baseline clinical data of the new-onset and persistent atrial fibrillation (PeAF)

Variables	PAF (n=83)	PeAF (n=43)	T or χ2	*P*-value
Age (year)	65.94±9.70	67.21±8.35	0.729	0.467
Gender (male)	45 (54.2%)	24 (55.8%)	0.029	0.864
BMI (kg/m2)	25.06±2.35	25.71±2.21	1.500	0.136
CHA2DS2-VASc	2.72±1.31	3.14±1.21	1.738	0.085
Hypertension	32 (38.6%)	20 (46.5%)	0.740	0.390
T2DM	30 (36.1%)	17 (39.5%)	0.139	0.709
HF	18 (21.7%)	17 (39.5%)	4.498	0.034
CAD	17 (20.5%)	14 (32.6%)	2.227	0.136
LAD (mm)	42.42±3.31	44.04±3.14	2.655	0.009
BNP (pg/ml)	65.62±7.34	70.46±8.30	3.354	0.001
TG (mmol/L)	2.57±0.41	2.55±0.44	0.266	0.790
TC (mmol/L)	6.03±0.43	6.00±0.49	0.224	0.823
LDL-C (mmol/L)	3.49±0.42	3.57±0.35	1.040	0.300
HDL-C (mmol/L)	1.79±0.34	1.69±0.35	1.562	0.121
MDA (mmol/ml)	7.11±0.96	7.66±0.72	3.618	<0.001
SOD (U/ml)	141.73±12.78	134.73±14.95	2.750	0.007
TNF-α (ng/ml)	58.27±6.69	61.30±6.45	2.439	0.016
IL-6 (pg/ml)	192.98±25.65	210.01±28.13	3.418	0.001
CTX-I (ng/ml)	1.34±0.16	1.48±0.15	4.707	<0.001
TGF-β1 (ng/ml)	18.67±2.60	20.29±3.25	3.044	0.003

**Table 3:** Correlations of serum irisin with clinical characteristics and biomarker levels of AF patients

	r	*P*-value
LAD (mm)	-0.184	0.039
BNP (pg/ml)	-0.387	<0.001
MDA (mmol/ml)	-0.186	0.037
SOD (U/ml)	0.206	0.021
TNF-α (ng/ml)	-0.194	0.030
IL-6 (pg/ml)	-0.222	0.013
CTX-I (ng/ml)	-0.288	0.001
TGF-β1 (ng/ml)	-0.262	0.003

Pearson correlation test was performed

## Discussion

This work investigated how irisin affected Ang II-induced AF and atrial fibrosis. Our research findings demonstrated that irisin expression was decreased in the serum of AF patients and mice treated with Ang II, whereas irisin inhibition had the opposite effects. Moreover, irisin substantially decreased atrial apoptosis; Ang II induced all atrial tissue inflammation, atrial fibrosis, and fibroblast transdifferentiation. Furthermore, irisin potentially suppresses the expression of LOXL2, TGF-β1, p-Smad2, and p-Smad3 in atrial tissue that is stimulated by Ang II. Therefore, the findings suggest irisin could be a potential target for the treatment of AF and atrial fibrosis.

We included 126 AF patients for the baseline study in the present study. Based on age and gender accumulation, we observed no potential difference between AF patients and healthy controls. However, high BMI, LAD, high expression of MDA, TNF-α, IL-6, CTX-I, TGF-β1, and reduced SOD level was found in the serum of AF patients in contrast to healthy controls (*P*<0.05). After classification of the AF patients into PAF and PeAF patients, we observed a high heart failure (HF) rate, increased levels of LAD, elevated serum concentrations of BNP, MDA, TNF-α, IL-6, CTX-I, TGF-β1, and lower expression of SOD (*P*<0.05) in PeAF patients than in PAF patients. Thus, the serum concentrations of BNP, MDA, TNF-α, IL-6, CTX-I, TGF-β1, and SOD, and levels of BMI and LAD might be a factor in the progression of atrial fibrosis as well as AF complications.

Several studies reported that in the adipose tissues, heart, and skeletal muscles, irisin is abundant (23, 24). A group of researchers demonstrated that the expression of irisin levels dropped in cardiac tissues and plasma after Transverse aortic constriction (TAC) and Ang-II-stimulated cardiomyocytes (25). Since the cardiac tissue is a plentiful irisin source (26), a loss of cardiomyocytes may reduce irisin after hypertrophy. One study shows that exogenous irisin may alter the NLRP3-stimulated inflammation (27), suggesting that exogenous irisin may potentially affect AF. Our study demonstrated lower levels of irisin expression in AF and PeAF patients. In addition, irisin treatment significantly reduces Ang II-induced AF in mice, apoptosis, and inflammation in atrial tissue, atrial fibrosis, and suppressed fibroblast transdifferentiation in atrial tissue. Moreover, using an Ang II-infused mice model showed clear evidence that irisin could combat Ang II-associated atrial fibrosis. Thus, irisin may be a promising therapeutic target for treating AF and atrial fibrosis linked to Ang II. 

TGF-β1 is a multifunctional cytokine that promotes epithelial-mesenchymal transitions (EMTs) (28). TGF-β1-induced EMT significantly influences cardiovascular fibrosis and hypertrophy (29, 30). Smad3, a protein that is downstream of TGF-β1, forms the TGF-1/Smad3 axis, a crucial target for preventing cardiac hypertrophy. Inactivation of the TGF-β1/Smad3 axis could suppress cardiac hypertrophy and suppress the transformation of the fibroblast to myofibroblast (31). Additionally, the TGF-β1/Smad3 axis increased the expression of connective tissue growth factor and caused the fibroblasts to produce collagen type I (32). In this current study results demonstrated the atrial tissue of mice with Ang II infusion enhanced the expression of LOXL2, TGFβ1, p-Smad2, and p-Smad3. The findings raised the possibility that activating the TGF-β/Smad pathway contributes to the development of atrial fibrosis.

Early research showed that in the LOX knockout mice, the connection of LOX and TGF-β signaling pathway results in the down-regulation of TGF expression in the bronchoalveolar lavage fluid (BALF) and a reduction in the proportion of pSmad2/3-positive cells in the pulmonary tissues (33). In addition, the development and progression of pulmonary fibrosis is influenced by LOXL2, and LOX may be connected to the TGF-β signaling pathway. The effects of LOXL2 on lung fibroblast proliferation and fibrosis development in mice with bleomycin (BLM)-mediated pulmonary fibrosis and the association of LOXL2 in the advancement of pulmonary fibrosis via the TGF-β1/Smad signaling pathway have not been adequately studied (34, 35). However, our current study showed that LOXL2, TGF-β1, p-Smad2, and p-Smad3 protein expression were significantly lower in the atrial tissue of mice injected with Ang II and irisin in contrast with those infused with Ang II only.

## Conclusion

We explored the potential effects of irisin on Ang II-induced AF and atrial fibrosis and its significant role in the LOXL2 and TGFβ1/Smad2/3 signaling pathways. Herein we reported that irisin attenuated atrial fibrosis brought on by Ang II, as well as AF vulnerability, through inactivation of LOXL2 and TGFβ1/Smad2/3 signaling pathway. Our findings speculate that irisin may serve as both a biomarker for diagnosing AF and a therapeutic target for treating atrial fibrosis and AF.

## Authors’ Contributions

ZN designed the project, supervised the project, and revised the manuscript. YW and JL performed experiments and wrote the first draft of the manuscript. XS, WG, and SW helped perform the experiments and collect data. SH and ZD analyzed the data and performed the statistical analysis. 

## Conflicts of Interest

The authors declare no conflicts of interest with other people or organizations.
